# BiFCROS: A Low-Background Fluorescence Repressor Operator System for Labeling of Genomic Loci

**DOI:** 10.1534/g3.117.040782

**Published:** 2017-04-25

**Authors:** Sarah Milbredt, Torsten Waldminghaus

**Affiliations:** Chromosome Biology Group, LOEWE Center for Synthetic Microbiology, SYNMIKRO, Philipps-Universität Marburg, D-35043 Marburg, Germany

**Keywords:** chromosome, segregation, microscopy, *Escherichia coli*, replicon

## Abstract

Fluorescence-based methods are widely used to analyze elementary cell processes such as DNA replication or chromosomal folding and segregation. Labeling DNA with a fluorescent protein allows the visualization of its temporal and spatial organization. One popular approach is FROS (fluorescence repressor operator system). This method specifically labels DNA *in vivo* through binding of a fusion of a fluorescent protein and a repressor protein to an operator array, which contains numerous copies of the repressor binding site integrated into the genomic site of interest. Bound fluorescent proteins are then visible as foci in microscopic analyses and can be distinguished from the background fluorescence caused by unbound fusion proteins. Even though this method is widely used, no attempt has been made so far to decrease the background fluorescence to facilitate analysis of the actual signal of interest. Here, we present a new method that greatly reduces the background signal of FROS. BiFCROS (Bimolecular Fluorescence Complementation and Repressor Operator System) is based on fusions of repressor proteins to halves of a split fluorescent protein. Binding to a hybrid FROS array results in fluorescence signals due to bimolecular fluorescence complementation. Only proteins bound to the hybrid FROS array fluoresce, greatly improving the signal to noise ratio compared to conventional FROS. We present the development of BiFCROS and discuss its potential to be used as a fast and single-cell readout for copy numbers of genetic loci.

The ability to visualize ever more details within organisms and cells has been a main driving force of biological science for many years, starting with the early microscopy studies of Anton van Leeuwenhoek. More recently, the discovery and application of fluorescent proteins have revolutionized life science, such as the small green fluorescent protein (GFP), the discovery of which was consequently honored with the Nobel Prize to Osamu Shimomura, Martin Chalfie, and Roger Tsien in 2008. Through genetic manipulation, the fluorescence can be added to individual proteins and then visualized by fluorescence microscopy or quantified by flow cytometry or other methods (Chalfie *et al.* 1994; Wei and Dai 2014). While this approach is straightforward for the analysis of proteins, other cellular components, for example DNA or lipids, are less easy to visualize. In particular, the labeling of DNA is of high importance since processes such as DNA replication or chromosome segregation and folding are central processes in all living cells (Messerschmidt and Waldminghaus 2014; Wang *et al.* 2008; Zickler and Kleckner 1999). Different methods have been developed to fluorescently label the entire DNA of a cell by, for example, using thymidine-analog incorporation or labeling of proteins that show uniform binding to DNA (Salic and Mitchison 2008; Wery *et al.* 2001). However, such approaches do not allow visualization of specific genetic loci. One approach to do so is Fluorescence *In situ* Hybridization (FISH), a method that uses sequence complementarity to target a genetic locus by hybridization with a fluorescent probe (DeLong *et al.* 1989; Scherthan and Loidl 2010). The disadvantage of FISH is that cells have to be fixed and therefore analysis of dynamics within living cells is not possible. An alternative approach is making use of the sequence-specific binding mediated by the bacterial CRISPR/Cas9 system. The short guide RNAs (sgRNAs) can be designed to target basically any sequence in a genome. This allowed visualization of repetitive sequences as telomeres based on a single sgRNA or nonrepetitive gene regions based on an array of many different sgRNAs (Chen *et al.* 2013). Fluorescent proteins are either fused to a dCas9 deficient in cutting DNA or to RNA-binding proteins to bind extended sgRNAs with respective target domains (Shao *et al.* 2016). Notably the latter allows dual labeling of genetic loci.

One popular alternative for labeling of genomic regions is the use of the so-called fluorescence repressor operator system (FROS) (Lau *et al.* 2003; Robinett *et al.* 1996), based on fusions between a fluorescent protein and a repressor that binds specifically to a respective operator sequence. An array of several such operator sequences is inserted at the locus of interest. This allows visual tracking of genetic loci by fluorescence microscopy because multiple labeled repressor proteins bound to the operator array appear as a fluorescence focus. FROS was initially applied with a LacI-GFP fusion that bound to an array of Lac operators in CHO and yeast cells (Gordon *et al.* 1997; Robinett *et al.* 1996). Additionally, FROS arrays were established based on the tet-repressor protein TetR and cI from the λ-phage (Fekete and Chattoraj 2005; Michaelis *et al.* 1997). FROS was successfully applied in various organisms to gain new insights into the localization, replication, and segregation of chromosomes in individual cells (Lau *et al.* 2003; Matzke *et al.* 2005; Straight *et al.* 1996). In addition to studies on the spatial and temporal organization of genetic loci, FROS can also be used to determine copy numbers of genetic loci, because the number of fluorescence foci should indicate the number of copies of the tagged locus (Jonas *et al.* 2011; Srivastava and Chattoraj 2007). A problem with this approach is that genetic loci do not necessarily have to be spatially separated but could be held together by, for example, sister chromosome cohesion (Lesterlin *et al.* 2012; Sherwood *et al.* 2010).

Generally, fluorescence-based analyses suffer from background fluorescence which reduces the true signal (Moolman *et al.* 2015). Weak signals can be superimposed by background fluorescence, so that false negative results may occur. Background fluorescence is an unspecific fluorescence signal that can be caused by various factors. Media, agents like antibiotics, unbound fluorophores, or fluorescence of the specimen itself can interfere with fluorescence analyses (Waters 2009). Moreover, cells always exhibit a natural intrinsic fluorescence, also known as autofluorescence, based on endogenous molecules that possess specific excitation and emission wavelengths, and which results in characteristic fluorescence spectra associated with specific species or strains (Ammor 2007). Background fluorescence can also be caused by experimental setup and imaging parameters, like light from the light source or camera noise (Joglekar *et al.* 2008). Different approaches can be used to solve the problem of high background signals. Examples are treatment with chemicals such as CuSO_4_ that disrupt background while signals remain, new microscopy techniques such as total internal reflection fluorescence microscopy, or application of mathematical models (Axelrod 2001; Schnell *et al.* 1999; Van de Lest *et al.* 1995). FROS also suffers from background fluorescence that is caused by the freely diffusing fluorescent proteins not bound to the operator array. Fluorescence microscopic analyses images arise with distinct foci paired with a diffuse fluorescence, which is distributed throughout the cell. Conceptually, elimination of this background should allow analysis of gene copy number in individual cells to be carried out simply by measuring the whole-cell fluorescence, for example, by flow cytometry.

Our current study describes the design and construction of a low-background hybrid FROS in *Escherichia coli*. It is based on the bimolecular fluorescence complementation (BiFC) of two repressor fusions binding to a respective hybrid FROS array. This new bimolecular fluorescence complementation and repressor operator system (BiFCROS) shows significantly less background fluorescence in comparison to conventional FROS. We also elucidated its ability to determine genetic copy numbers by measurement of whole-cell fluorescence.

## Materials and Methods

### Bacterial strains, plasmids, oligonucleotides, and culture conditions

All strains, replicons, and oligonucleotides used in this study are listed in Supplemental Material, Tables S1–S3 in File S1. Bacteria were cultured in Luria-Bertani (LB) liquid medium under vigorous shaking or on LB plates at 37° with the desired antibiotic if not indicated otherwise. Antibiotic selections were used at the following concentrations: 100 μg/ml ampicillin, 12.5 μg/ml chloramphenicol, 35 μg/ml kanamycin, and 15 µg/ml tetracycline.

### Fluorescence microscopy

For fluorescence microscopy, cells were grown in AB-glucose or AB-sodium acetate until OD_450_ ∼0.15 (Messerschmidt *et al.* 2015). Expression of fusion proteins was induced with 400 nM isopropylthio-β-galactoside for 1 hr in AB-glucose and 2 hr in AB-sodium acetate. One milliliter of culture was harvested by centrifugation, the supernatant was discarded, and cells were resuspended in 20 µl of the same medium, then 1.5 µl of cells were transferred to 1% agarose pads based on respective growth medium.

Fluorescence microscopy was performed with a Nikon Eclipse Ti-E microscope with a phase-contrast Plan Apo l oil objective (100; numerical aperture, 1.45) with the AHF HC filter set F36-528 YFP [excitation band pass (ex bp) 500/24-nm, beam splitter (bs) 515-nm, and emission (em) bp 535/30-nm filters] and an Argon Ion Laser (Melles Griot). Exposure times were adjusted for each sample. Images were acquired with an Andor iXon3 885 electron-multiplying charge-coupled device camera. Images were analyzed with Fiji using the plugin MicrobeJ (Jiang *et al.* 2014). Data were further processed with Excel and R statistics.

### Construction of the OL1/UAS hybrid FROS array

**Figure 1:**
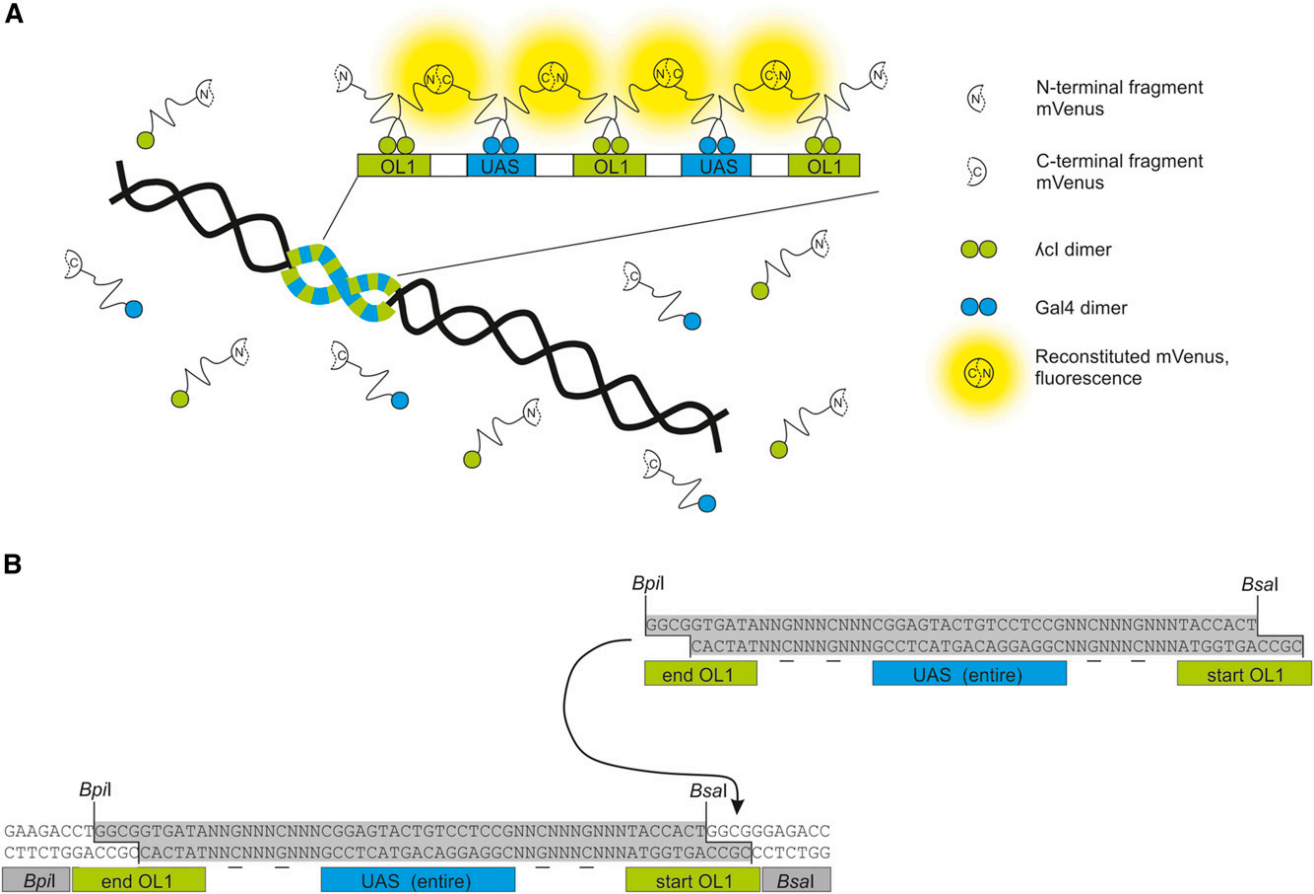
Scheme of BiFCROS, an improved fluorescence repressor operator system. (A) An array with alternating binding sites OL1 and UAS is integrated into the *E. coli* chromosome. Respective proteins λcI and Gal4 are fused to either the N- or C-terminal fragment of a fluorescence reporter (mVenus). Through binding of the proteins to the array, terminal fragments of mVenus come in close proximity and reconstitute resulting in a fluorescence signal. Proteins that are not bound do not fluoresce and cause no background signal. (B) Initial building blocks of the operator array contain a split OL1 site, an entire UAS and recognition sequences for type IIs restriction enzymes *Bpi*I and *Bsa*I. Random spacer sequences between binding sites contain two determined base pairs (underlined). Restriction digestion with *Bpi*I and *Bsa*I results in DNA fragments with overhangs fitting to each other (highlighted in gray). Ligation of two DNA fragments leads to a full OL1 site.

An initial DNA building block was designed containing an UAS and a split OL1 binding site for hierarchical assembly of the final array (Figure 1B). For generation of the DNA fragment, oligonucleotides 257 and 258 were annealed resulting in single stranded 5′ overhangs for ligation into an *Xba*I and *EcoR*I-cut pUC19 and pUC57-kan. Ligation products were transformed into *E. coli* XL1-Blue and all colonies were used as the pool for plasmid isolation. Resulting plasmid libraries were divided into two parts which (i) were cut with *Bpi*I and *Nde*I (vector) and (ii) were cut with *Bsa*I and *Nde*I (insert). pUC57-insert was ligated with pUC19-vector and vice versa. Ligation products were transformed into *E. coli* XL1-Blue and should have contained combinations of two initial inserts. Cycles from plasmid library isolation, restriction digestion, and ligation were repeated until 64 inserts were assembled. One final clone was chosen and the construct sequenced to verify length and accuracy of OL1 and UAS binding sites. The plasmid was named pMA164. It is noteworthy that we have meanwhile developed a faster and more efficient way to assemble respective FROS arrays (Schindler *et al.* 2016). For integration into the *E. coli* chromosome, 500 bp of the upstream (primer 95 + 96) and downstream (primer 97 + 98) region of *tnaA* were amplified. Together with *Sma*I and *Dra*I-cut pMA164, the amplicons were used in a Gibson assembly (Gibson *et al.* 2009) that resulted in plasmid pMA165. In the next step, the origin of replication of pMA165 was exchanged. A conditional origin of replication, *oriR6K*, was amplified from synVicII-0.1 (Messerschmidt *et al.* 2015) with flanking *Sma*I recognition sites (primer 389 + 390). Via Gibson assembly, the *oriR6K* fragment and pMA165, which was cut with *Sma*I, were assembled, resulting in pMA252. pMA252 was cut with *Sma*I and used for integration into the *E. coli* chromosome via lambda red recombination using *E. coli* strain D50 (Datsenko and Wanner 2000). Integration was first verified by colony PCR (primer 102 + 103) and then by Southern blot analysis with a DIG labeled *mnmE* specific probe [409 + 410; PCR DIG probe synthesis kit (Roche; data not shown)]. The integrated array was transferred to *E. coli* MG1655 via P1 transduction.

### Construction of fusion proteins

All fusion constructs of λcI and Gal4 to mVenus were done by Gibson assembly (Gibson *et al.* 2009). For λcI fusions, an RSIAT (cgcagcattgcgacc) linker was integrated either between λcI and mVenus, or the N-terminal fragment of mVenus. Fusion constructs that included the Gal4 DNA-binding domain (amino acids 1–441) of *Saccharomyces cerevisiae* contained no linker. BiFC constructs contained a random linker sequence of 21 bp between λcI fused to the N-terminal fragment of mVenus (λcI-VN) and Gal4 fused to the C-terminal fragment of mVenus (Gal4-VC). The mVenus amino acids 1–154 were used as the N-terminal fragment and amino acids 155–238 as the C-terminal fragment. For improved signal to noise ratio, mutation I152L was introduced in the N-terminal fragment of mVenus by PCR (Kodama and Hu 2010). Resulting plasmids were verified by restriction digestion and sequencing. A detailed description of the preparation of different DNA fragments for Gibson assembly is listed in Table S4 in File S1.

### Construction of replicons with and without OL1/UAS hybrid FROS array

The ampicillin cassette of the synthetic chromosome synVicII-1.3 was exchanged with a chloramphenicol cassette (primer 655 + 657) by homologous recombination in yeast (Messerschmidt *et al.* 2015). The resulting replicon was named pMA182. For insertion of the OL1/UAS hybrid FROS array into pMA182, it was amplified with primer 1045 + 1046 and pMA182 was cut with I-SceI and *Nhe*I. Both products were used in a Gibson reaction to assemble plasmid pMA301. pMA182 naturally contains a *gfp* which would interfere with mVenus fluorescence signals. *gfp* was removed by cutting of pMA182 with *I-Sce*I and *Nhe*I and ligated with annealed primer 1259 + 1260 resulting in pMA310.

### Data analysis with MicrobeJ and R statistics

The plugin MicrobeJ for Fiji was used to detect raw pixel intensities and intensities of whole cells. Data were imported into Excel and and R custom scripts were used for further analysis.

### Data availability

Strains are available upon request. Figure S1 in File S1 shows biological replicate of data shown in Figure 5. Tables S1–S3 in File S1 provide details of strains, replicons, and oligonucleotides used in this study. Table S4 in File S1 gives details on primer and templates used for construction of plasmids.

## Results

### BiFCROS

The background fluorescence in FROS systems is caused by the non-DNA-bound fluorescence repressor fusions. Therefore, it should be possible to reduce the background signal by reducing the freely diffusing fluorescent proteins. To this end, we made use of the ability of split fluorescent proteins to complement one another if in close spatial proximity (Kerppola 2008). The general logic of our new approach is illustrated in Figure 1A. Instead of one transcriptional repressor protein fused to a full-length fluorescent protein, the new system consists of two repressor proteins (λcI and Gal4), each fused to one half of the fluorescent protein mVenus. These protein fusions should not generate any fluorescence signal unless they come into close spatial proximity. Such a signal would only be desirable at a genetic locus of interest. To this end, we constructed a hybrid array of operator sites with alternating binding sites for λcI (OL1) and Gal4 (UAS) (Figure 1A). The design includes 64 instances of each operator type and was constructed with a seven-step hierarchical cloning scheme based on libraries of synthetic DNA oligos and type IIS restriction enzymes (Figure 1B; see *Materials and Methods* section for details).

### Low-background signals with BiFCROS

**Figure 2:**
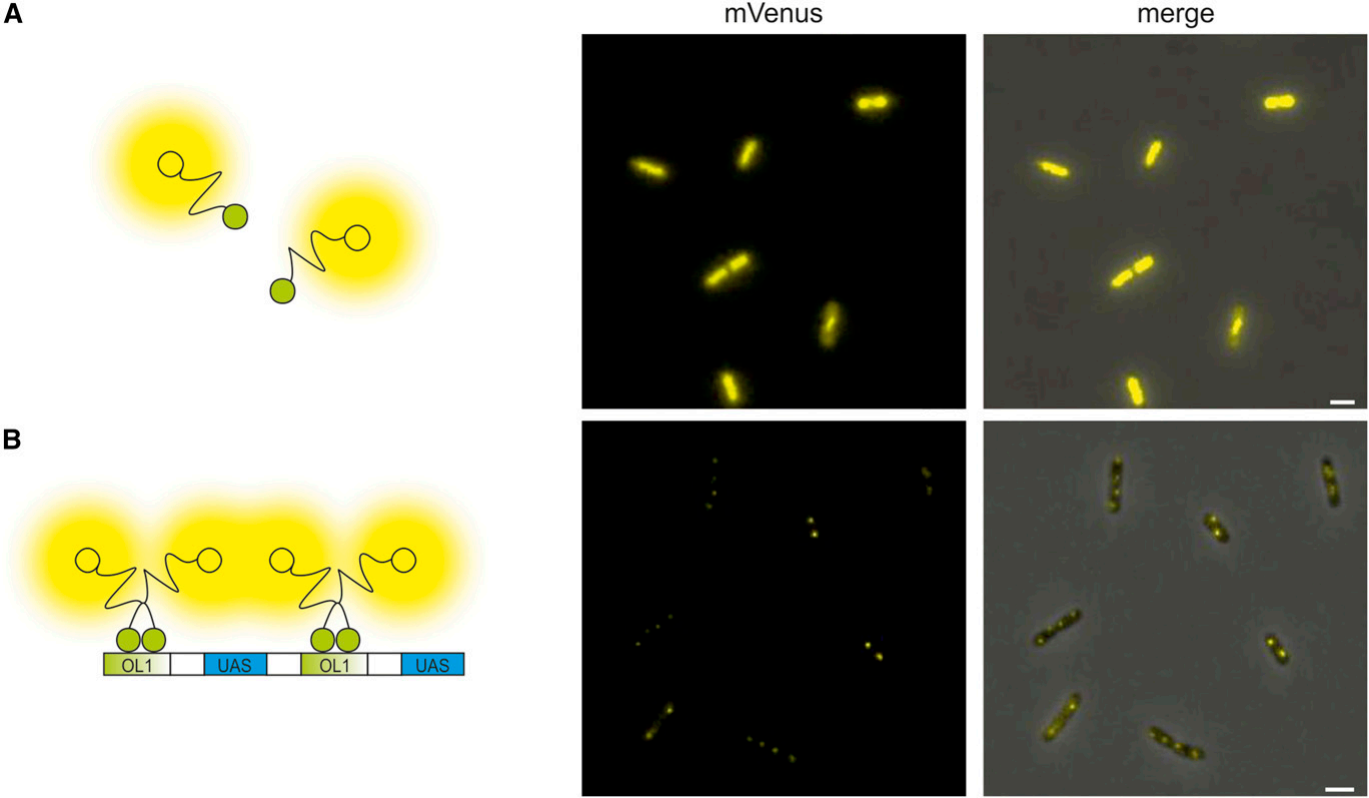
Specific binding to a hybrid FROS array. Fluorescence microscopy images of cells without (A, strain SM69) and with (B, strain SM77) OL1/UAS hybrid FROS array expressing a fusion of the λcI repressor to full-length mVenus. Left panel presents cartoons illustrating fluorescent fusion proteins in respective *E. coli* strains. Fluorescence microscopy images show the mVenus channel (middle panel) or merges of mVenus and phase-contrast channel (right panel). Exposure time was set to 100 msec. Bar, 2 µm.

**Figure 3:**
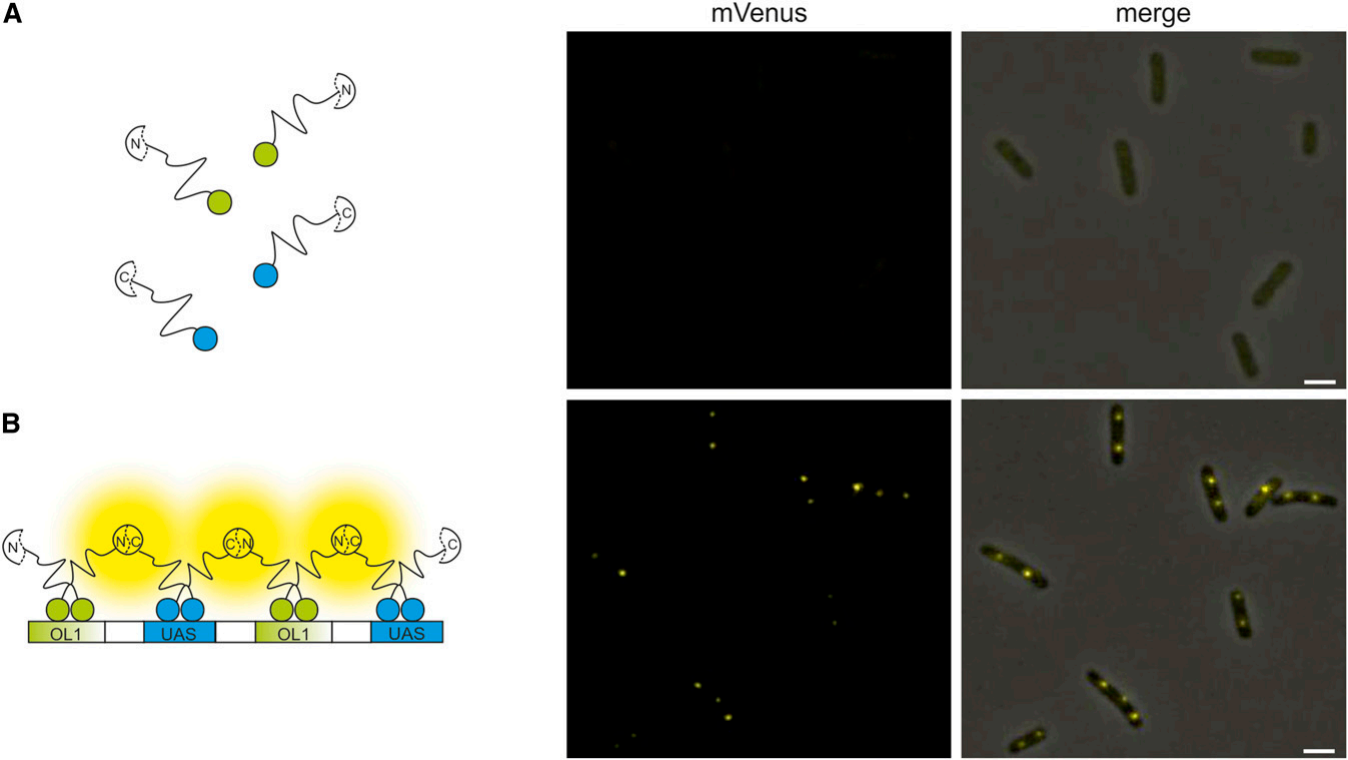
Bimolecular complementation of split mVenus mediated by binding of fusion proteins to a hybrid FROS array. Fluorescence microscopy images of cells that express BiFC constructs of λcI fused to the N-terminal fragment of mVenus and Gal4 fused to the C-terminal fragment of mVenus without (A, strain SM57) and with OL1/UAS hybrid FROS array (B, strain SM65). Left panel presents cartoons illustrating fluorescent fusion proteins in respective *E. coli* strains. Fluorescence microscopy images show the mVenus channel (middle panel) or merges of mVenus and phase-contrast channel (right panel). Exposure time was set to 200 msec. Bar, 2 µm.

As a first test of array functionality we constructed an inducible system for expression of a full-length mVenus fused to the λcI repressor. Expression of this fusion-protein in *E. coli* cells without the constructed hybrid FROS array resulted in diffuse fluorescence as expected (Figure 2A). In contrast, clear fluorescence foci were visible when cells contained the OL1/UAS hybrid FROS array, showing that the array indeed binds the protein fusion (Figure 2B). To test the BiFCROS principle outlined above, we fused the λcI repressor to the N-terminal half of mVenus and Gal4 to the C-terminal part within an inducible bicistronic operon under an inducible promoter. *E. coli* cells expressing these fusion proteins showed very low fluorescence and no distinct foci (Figure 3A). In contrast, clear fluorescence foci were detected in cells with a chromosomal insertion of the OL1/UAS hybrid FROS array (Figure 3B). These results indicated that the hybrid FROS array is in fact able to mediate fluorescence complementation and limits the fluorescence signal to the targeted genetic locus.

**Figure 4:**
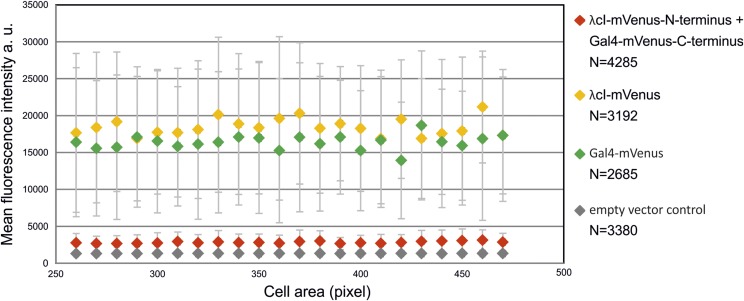
Low background fluorescence in cells with BiFC constructs without hybrid FROS array. Mean fluorescence intensities of binned cells *vs.* cell area were plotted as indicated. Strain SM57 (red) contained λcI fused to the N-terminal fragment of mVenus and Gal4 fused to the C-terminal fragment of mVenus, strain SM69 (yellow) contained λcI fused to full-length mVenus, strain SM70 (green) contained Gal4 fused to full-length mVenus, and strain SM126 (gray) contained the empty vector. Fluorescence microscopy was performed with cells grown exponentially in AB-glucose. Exposure time was set to 100 msec. Number of recorded cells is indicated (N). Quantification of fluorescence signals was done by MicrobeJ and data analyses by R. Cells were grouped according to their cell area with moving windows of 10 pixels. Groups with <10 cells were excluded. Average values of two independent experiments are shown.

**Figure 5:**
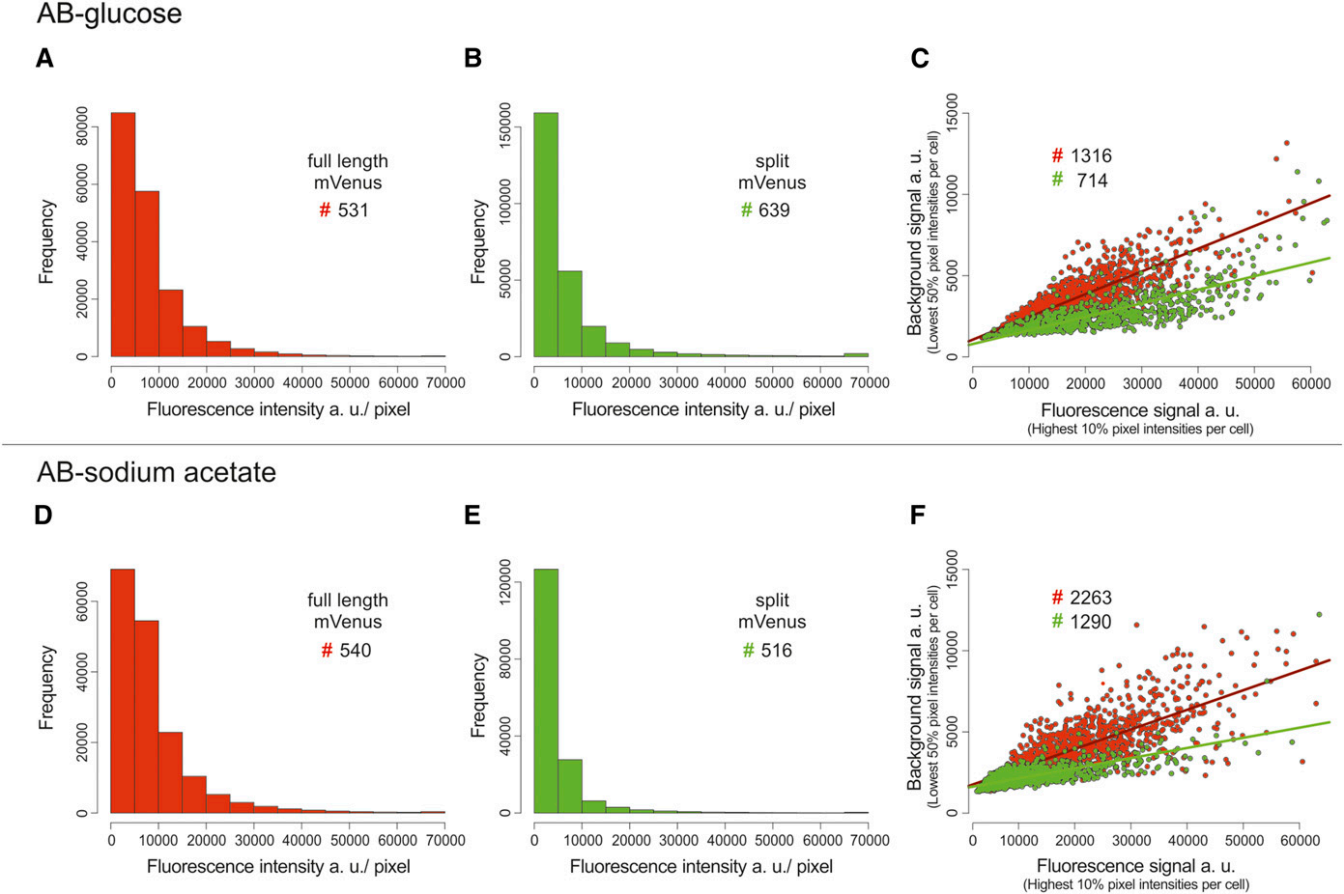
Improved signal to background with split mVenus compared to full-length mVenus. Cells with OL1/UAS hybrid FROS array and λcI fused to full-length mVenus (highlighted in red, strain SM77) or BiFC constructs λcI fused to the N-terminal fragment of mVenus and Gal4 fused to the C-terminal fragment of mVenus (highlighted in green, strain SM65) were analyzed under fast growth (top panel, AB-glucose) or slow growth conditions (bottom panel, AB-sodium acetate). (A, B, D, and E) Frequency distribution of pixel fluorescence intensities from several hundred cells as indicated by #. Only cells with intensities below 65,000 and >fivefold signal to background ratio were analyzed [mean of 10% pixels with highest (signal)/ mean of 50% pixels with lowest intensity (background)]. (C and F) Scatter plots of mean fluorescence intensity of the highest 10% pixels *vs.* mean fluorescence intensity of the lowest 50% pixels within individual cells. Values above 65,000 on *x*-axis and above 15,000 on *y*-axis were excluded. Exposure times in AB-glucose were 50 msec for full-length mVenus and 200 msec for split mVenus and for AB-sodium acetate 50 msec for full-length mVenus and 600 msec for split mVenus. Quantification of fluorescence signals was done by MicrobeJ and data analyses by R with custom R scripts.

To get a more quantitative insight into background intensities, we quantified the fluorescence per cell for *E. coli* strains with (i) the BiFC constructs with λcI and Gal4 each fused to one part of the split mVenus (strain SM57); (ii) λcI fused to the full-length mVenus (strain SM69); (iii) Gal4 fused to a full-length mVenus; and (iv) an empty-vector control, respectively (Figure 4). Cells were grouped according to their size and average values of fluorescence calculated for each bin. The analysis shows that splitting of mVenus greatly decreases the total cellular fluorescence by almost one order of magnitude compared to the full-length fusion constructs (Figure 4). The fluorescence of the split fusion constructs was only slightly higher than the negative control (empty vector). More important than the general reduction of background fluorescence by the fluorophore splitting approach was an increase of the signal to background ratio. Considering pixels of individual cells, one would expect many pixels of very low intensity and a small fraction of pixels with high intensities corresponding to the fluorescence foci. Pixels with intermediate intensities should be rare in a low-background system. To measure these values for BiFCROS, we performed quantitative fluorescence microscopy of cells with the hybrid FROS array and either the split fusion constructs or a fusion of λcI to the full-length mVenus (Figure 5). Cells were grown in different growth media resulting in either fast (AB-glucose) or slow growth (AB-sodium acetate). Fluorescence intensity values for individual pixels of some hundred cells were calculated and plotted as histograms (Figure 5, A, B, D, and E), the shapes of which were clearly different. In cells with BiFCROS, most pixels belonged to the lowest intensity fraction, with a sharp drop in pixel numbers belonging to fractions with higher intensities (Figure 5, B and E). In cells with the full-length mVenus fusion, comprising the conventional FROS system, this drop was much less pronounced with many pixels comprising intermediate fluorescence intensity (Figure 5, A and D). These results indicate that the distribution of background to signal fluorescence is much clearer with BiFCROS compared to conventional FROS. To analyze the ratio of signal to background for individual cells, we defined the signal as 10% of the pixels with the highest fluorescence intensity and the background as the 50% with the lowest signal. Respective mean values of signal and background were plotted *vs.* one another for individual cells (Figure 5, C and F). Biological replicates of the analysis showed good reproducibility (Figure S1 in File S1). The plots showed an increase of background with increasing signal intensity for the full-length mVenus fusion, comprising the conventional FROS system (Figure 5, C and F; red dots and regression line). A similar correlation was found for the BiFCROS approach (Figure 5, C and F; green dots and regression line). However, the slope of the respective regression line was much flatter compared to the conventional FROS system, indicating an improved signal to background ratio within individual cells carrying BiFCROS.

### Toward copy number determination with BiFCROS

**Figure 6:**
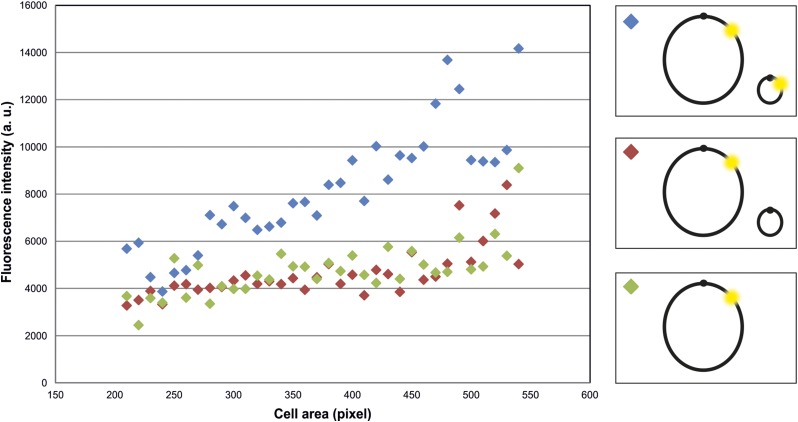
Fluorescence signals of cells with one or two copies of the hybrid FROS array. Mean fluorescence intensities of cells were binned and plotted *vs.* cell area. *E. coli* strains used all have a copy of the OL1/UAS hybrid FROS array on the primary chromosome as depicted in the scheme (left panel). Strain SM153 (blue) carries replicon pMA301 with an additional copy of the OL1/UAS hybrid FROS array while the additional replicon (pMA310) in strain SM158 (red) presents the respective empty-vector control. Strain SM65 (green) contains only the BiFCROS array without additional replicon. All strains contain BiFC constructs of λcI fused to the N-terminal fragment of mVenus and Gal4 fused to the C-terminal fragment of mVenus. Fluorescence microscopy was performed with cells grown in AB-sodium acetate medium. Exposure time was set to 600 msec. Approximately 1000 cells per strain were recorded. Quantification of fluorescence signals was done by MicrobeJ and data analyses by R. Mean intensity of cells of strain SM57 that contains BiFC constructs with split mVenus without OL1/UAS hybrid FROS array was determined as background signal and subtracted from cell intensities of strains shown here. Cells were grouped according to their cell area in bins of 10 pixels. Groups with <10 cells were excluded. Average values of three independent experiments are shown. SD was not plotted for clarity reasons.

**Figure 7:**
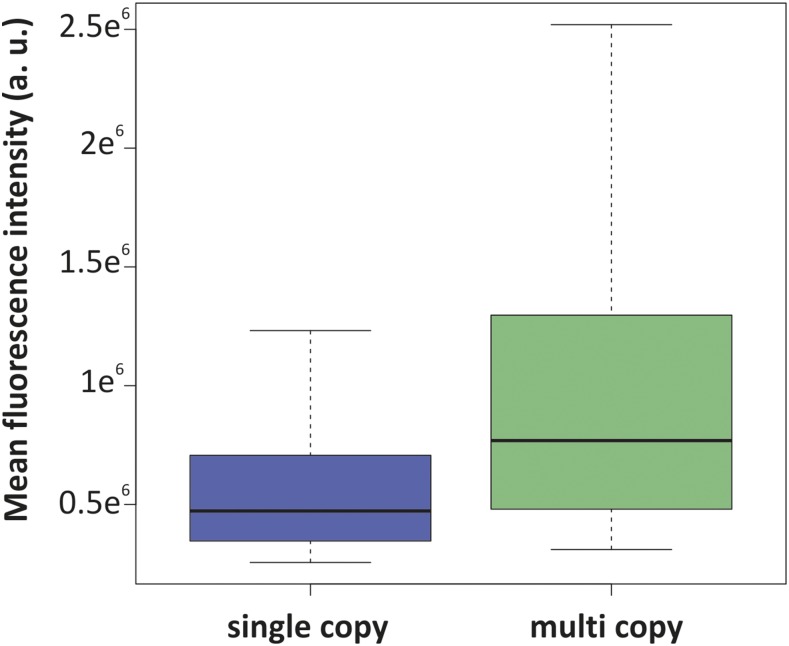
Fluorescence signals of cells with one or multi copies of the hybrid FROS array. Mean fluorescence intensities of cells with the 50% highest fluorescence intensity are shown for an *E. coli* strain carrying a single copy of the OL1/UAS hybrid FROS array on the primary chromosome (strain SM65, blue, *N* = 239) or the array on a multicopy plasmid (strain SM68, green, *N* = 1050). Both strains contain BiFC constructs of λcI fused to the N-terminal fragment of mVenus and Gal4 fused to the C-terminal fragment of mVenus. Fluorescence microscopy was performed with cells grown in AB-sodium acetate medium. Exposure time was set to 50 msec. Quantification of fluorescence signals was done by MicrobeJ and data analyses by R. Mean intensity of cells of strain SM57 was determined as background signal and subtracted from intensities of cells shown here.

The cellular copy number of genetic loci is an important characteristic and can, for example, provide information about the replication status of cells, with a duplicated copy number indicating completed DNA replication. The design of BiFCROS should in principle allow simple and single-cell copy number determination of gene loci. Since only the array-bound split proteins give a signal, the number of hybrid FROS array copies should be proportional to total fluorescence intensity of the cell. Determination of the fluorescence intensity of the whole cell, for example, by flow cytometry could thus be used to measure genetic copy numbers. To test this assumption, an experimental set up was generated with a strain that contained one copy (SM65) and a strain which contained two copies of the OL1/UAS hybrid FROS array (SM153). The second copy of the array was embedded on an additional synthetic, secondary chromosome pMA301 (Messerschmidt *et al.* 2015, 2016). Fluorescence microscopy measurements of the two strains revealed significantly increased fluorescence intensity for the strain with two copies of the array compared to the intensity of the strain with only one copy (Figure 6). To rule out that the synthetic chromosome contributed to the fluorescence in any way, a control strain was generated that contained the array on the native chromosome and additionally carried a synthetic, secondary chromosome without a BiFCROS array (SM158). The fluorescence intensities of this strain were comparable to the strain that carries no additional replicon (Figure 6). A limitation of the BiFCROS system might occur when extremely different copy numbers are compared. This is because too many BiFCROS arrays relative to the number of split fluorescent proteins will lead to low binding densities per array and thus low output signals. To test this hypothesis we analyzed a strain carrying the BiFCROS array on a multicopy plasmid and compared it to a strain with a single chromosomal insertion (Figure 7). Indeed, the mean fluorescence increased only ∼1.7-fold comparing single to multicopy, indicating that the BiFCROS system is not well suited to measure extreme copy number variations.

## Discussion

Observation of spatial and temporal organization of the DNA within the cell is important to gain insights into cellular processes like DNA replication or chromosome segregation. In any measuring system, an optimal signal to background ratio is desirable. Although FROS is a commonly used application to highlight DNA *in vivo*, no attempt has been made until now to reduce its background fluorescence. Here, we present BiFCROS, a new single-cell method that has a strongly reduced background signal. It is based on BiFC, the ability of a fluorescent protein to be fragmented and complement if in close proximity (Figure 1). BiFC has been used previously to fluorescently label DNA *in vitro* (Stains *et al.* 2005). Stains and coworkers developed a tool for direct targeting of double-stranded DNA sequences, called SEER (SEquence Enabled Reassembly of proteins). They used a split GFP, fused to two different Zinc finger domains, that reconstituted upon binding to designated DNA *in vitro* (Stains *et al.* 2005). BiFC was also applied to visualize and track RNA in living cells in eukaryotes, where RNA is more readily accessible than DNA, which is located in the nucleus (Valencia-Burton *et al.* 2007). The method was based on fluorescence complementation regulated by the interaction of a split RNA-binding protein with its corresponding RNA aptamer (Valencia-Burton *et al.* 2007). In *E. coli*, this system was applied to study temporal and spatial organization of RNA (Valencia-Burton *et al.* 2009). Interestingly, the study showed less fluorescence background signals in comparison to analysis with full-length fluorescent proteins as found in the work presented here.

Different fluorescent proteins have been used in complementation experiments. mVenus was chosen for BiFCROS because it has a fast and efficient maturation time, is less sensitive to the environment compared to other fluorescent proteins, and Venus-based BiFC showed 10-fold higher fluorescence intensity in comparison with signals from, *e.g.*, eYFP-BiFC (Nagai *et al.* 2002; Shyu *et al.* 2006). mVenus fusions were constructed with a monomeric variant to prevent dimer formation and to exclude the resulting fluorescence signal from the latter. For further improvement of the signal to noise ratio, we inserted the amino acid exchange mutation I152L into the N-terminus of mVenus, which reduces spontaneous self-assembly of the nonfluorescent C- and N-terminal fragments of mVenus (Kodama and Hu 2010) and increases the signal to noise ratio fourfold in BiFC assays in comparison to other mVenus variants. One disadvantage of using mVenus is that it is known to emit in wavelength regions in which the autofluorescence is very high (Telford *et al.* 2012). A potential way to optimize BiFCROS performance in the future could thus be using a BiFC-compatible fluorescence protein that emits in the red range, such as dsRed and its improved derivatives, *e.g.*, mRFP1 (Knop *et al.* 2002). mRFP1 has already been applied successfully in BiFC analyses (Jach *et al.* 2006).

We have probed the potential of whole-cell fluorescence in BiFCROS cells as readout for genetic copy numbers (Figure 6 and Figure 7). Measuring the whole-cell fluorescence can be performed precisely and in high throughput by flow cytometry on a single-cell level. This would be a great advance compared to conventional copy number measurements, such as quantitative PCR or marker frequency analysis by microarrays or next-generation sequencing (Milbredt *et al.* 2016; Skovgaard *et al.* 2011; Stokke *et al.* 2011). In our experiments, fluorescence intensities of cells with two copies of the OL1/UAS hybrid FROS array were higher than cells with only a single copy (Figure 6). This observation could be explained in two alternative ways. First, the measured fluorescence could result exclusively from the bound complemented fusion proteins as desired. An alternative explanation, however, is that the hybrid BiFCROS array works as an assembly platform where the fusion proteins bind, complement, and leave the DNA while still producing a fluorescence signal. The latter explanation is supported by the observation that the whole-cell fluorescence in cells with BiFCROS increases linearly with increasing cell size (data not shown). If the first explanation would hold true, the whole-cell fluorescence should be biphasic relative to the cell size as a measure of the cell cycle stage, with a doubling at the time point of locus replication. Most probably, the fluorescence we observe is a mixture of DNA-bound complemented proteins and those which left the DNA but remained complemented. It is also important to consider the time required for fluorophore maturation, which might “smear out” the effect of sudden events such as replication of the respective genetic locus (Kerppola 2006). Another problem appears from the need for a good ratio of split fluorescent protein concentration and BiFCROS array concentration. With too many split proteins the likelihood of random reconstitution in the cytoplasm and with it the background fluorescence will increase. Too high a number of arrays will lead to each individual array not being saturated. This effect might explain the relatively low fluorescence in a strain carrying the BiFCROS array on a multicopy plasmid (Figure 7). Clearly, further optimizations are required to develop BiFCROS into a robust system for genetic copy number analysis for which we lay the basis here.

## Supplementary Material

Supplemental material is available online at www.g3journal.org/lookup/suppl/doi:10.1534/g3.117.040782/-/DC1.
