# Supplementary material for: BiFCROS: A Low-Background Fluorescence Repressor Operator System for Labeling of Genomic Loci
Source: G3 (Bethesda). 2017 Apr 25;7(6):1969–77. doi: 10.1534/g3.117.040782 (PMC5473772; doi:10.1534/g3.117.040782)
Supplement: Supplementary file 1 [file 1969FileS1.pdf]

Supporting information

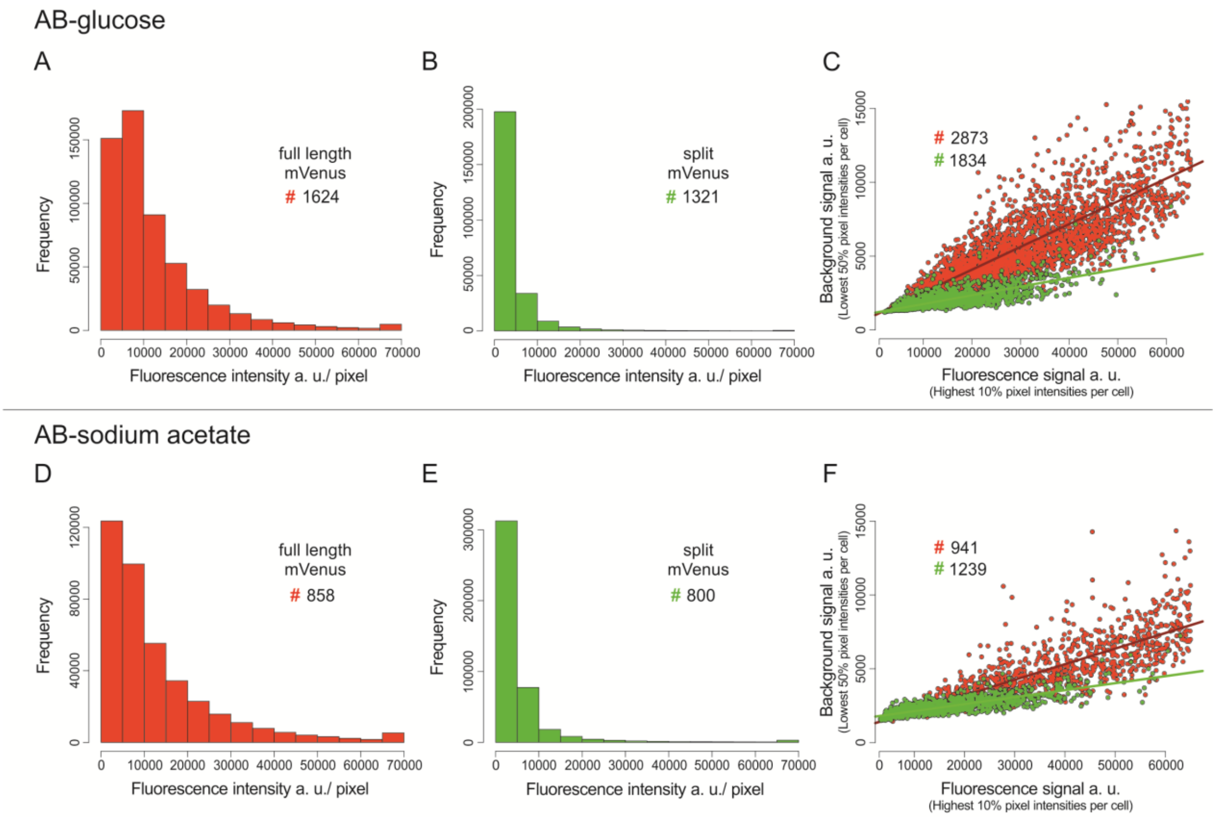

Figure S1: Biological replicate of data shown in figure 5 of the main article (compare respective figure legend).

Table S1: Strains used in this study.

Strain	Relevant genotype and additional characteristics	Resistance	Reference
E. coli XL1-Blue	*recA1 endA1 gyrA96 thi-1 hsdR17 supE44 relA1 lac*[F´ *proAB lacIqZ*ΔM15 *Tn10* (TetR)]	tetracycline, nalidixic acid	Stratagene
E. coli D50	BW25113 + pKD46	ampicillin	(Datsenko and Wanner, 2000)
E. coli MG1655	Wild type	-	(Blattner et al., 1997)
S. cerevisiae PJ69-4a	MAT*a, trp*1-901*, leu*2-3*, leu*2-112*, ura*3-52*, his*3-200*, gal4Δ, gal80Δ,* GAL2-ADE2, LYS2::GAL1HIS3*, met*2::GAL7-lac		(James et al., 1996)
SM29	MG1655, tnaA(74-1319)::OL1/UAS hybrid FROS array, kan^{R}	kanamycin	This study
SM57	MG1655 + pMA270	ampicillin	This study
SM65	SM29 + pMA270	kanamycin, ampicillin	This study
SM68	MG1655 + pMA165 + pMA270	kanamycin, ampicillin	This study
SM69	MG1655 + pMA275	ampicillin	This study
SM70	MG1655 + pMA276	ampicillin	This study
SM77	SM29 + pMA275	kanamycin, ampicillin	This study
SM126	MG1655 + pWM2060	ampicillin	This study
SM153	SM29 + pMA301 + pMA270	kanamycin, chloramphenicol, ampicillin	This study
SM158	SM29 + pMA310 + pMA270	kanamycin, chloramphenicol, ampicillin	This study

Table S2: Replicons used in this study.

Plasmid	Characteristics	Resistance	Reference
pKD46	*bla, ara, oriR101,* $rep101^{ts}$	ampicillin	(Datsenko and Wanner, 2000)
pUC57-kan	Cloning vector	kanamycin	GenScript, Piscataway Township, NJ, USA
pUC19	Cloning vector	ampicillin	Anke Becker
pPS19	pDS132, P$_{BAD}$ *lacI-eyfp λcI-ecfp* P$_{CP18}$*araE tetR*	chloramphenicol, spectinomycin	Dhruba Chattoraj (Srivastava et al., 2006)
pGBKT7	*E. coli / S. cerevisiae* Shuttle Y2H vector	kanamycin	Clonetech
pBad24-LacI-venus	*venus* fluorophor	ampicillin	(Hammar et al., 2014)
synVicII-0.1	*oriR6K, 2µ ori, URA-3*	ampicillin	(Messerschmidt et al., 2015)
synVicII-1.3	synVicII-1.0 P$_{A1/04/03}$-RBSII-*gfp*(AAV)	ampicillin	(Messerschmidt et al., 2015)
pWM2060	pTrc99A , promoter	ampicillin	Provided by W.

	down mutations in -35 and -10		Margolin (Bernard et al., 2007)
pMA164	pUC57 kan + OL1/UAS hybrid FROS array	kanamycin	This study
pMA165	pMA164 + tnaA-OL1/UAS hybrid FROS array-tnaA	kanamycin	This study
pMA182	synVicII-1.3, amp::cat	chloramphenicol	This study
pMA252	pMA164, *ColE1*::*oriR6K*	kanamycin	This study
pMA270	pWM2060 with λcI fused to N-terminal fragment of mVenus, Gal4 fused to C-terminal fragment of mVenus	ampicillin	This study
pMA275	pWM2060, λcI fused to full length mVenus	ampicillin	This study
pMA276	pWM2060, Gal4 fused to full length mVenus	ampicillin	This study
pMA301	pMA182, *gfp*::OL1/UAS hybrid FROS array	chloramphenicol	This study
pMA310	pMA182 Δ*gfp*	chloramphenicol	This study

Table S3: Oligonucleotides used in this study.

Name	Sequence
46	GTCTATCTGACCGCCGACAAGCAGAAGAAC
47	GTTCTTCTGCTTGTCGGCGGTCAGATAGAC
95	ACGCCAAGCTTGCATGCAGGCCTCTGCAGTCGACGGGCCCGGGAC CGGCAAGATCAACAGGTA
96	TCATGAGATTATCAAAAAGGATCTTCACCTAGATCCTTTTCCCGGGTAGCCATCACCAGAGCCAAA
97	GGTTAATTGGTTGTAACATTATTCAGATTGGGCTTGATTTCGCGAGTGGTACGTTTTACT
98	CAGTCATACCACTGGCGGGAGACCTCTAGATATCGGATCCCACATGTGAAAGAGAACGCGG
102	GCGAGGATAAGTGCATTATG
103	ACAATAACCCGGAATGAAGC
257	AATTCGAAGACCTGGCGGTGATANNGNNNCNNNCGGAGTACTGTCCTCCGNNCNNNGNNNTAC CACTGGCGGGAGACCT
258	CTAGAGGTCTCCCGCCAGTGGTANNNCNNNGNNCGGAGGACAGTACTCCGNNNGNNNCNNTAT CACCGCCAGGTCTTCG
267	ACAGCTCCTCGCCCTTGCTCACCATGGTCGCAATGCTGCGGCCAAACGTCTCTTCAGGCC
268	CGCAGCATTGCGACCATGGTGAGCAAGGGCGAGGAGCTGTTCAC
270	CAACTACAACAGCCACAACGTCTATCTGACCGCCTAACCCTAGCAGGAGGAATTCACCATGAAGCT ACTGTCTTCTATC
389	GTATGTTTGCTTTACCTGTTGATCTTGCCGGTCCCGGGAACCTGTTGATAGTACGTACTAAGC
390	GAATCTAATCGGTTTGGCTCTGGTGATGGCTACCCGGGCCATGTCAGCCGTTAAGTGTTCC
409	CTACAACAGGGCAAAGCGCAAC
410	CAAATCAAGGGCGGTGATCGAC
646	ATGGAATTCGAGCTCGGTACCCGCGTTCTAGCAGGAAACAGCTATG
655	TATATGAGTAAACTTGGTCTGACAGTCATCGCAGTACTGTTGTATTCATT
656	CTGCAGGTCGACTCTAGAGGATCCCCTTACTTGTACAGCTCGTCCATGCC
657	CCTGACTCAGTGCTTTCTATCCCGCTTTTGGCGAAAATGAGACG

712	GGTGAAAACCTCTGACACATGCAGCTTTACTTGTACAGCTCGTCCATGCCGAGA
714	GTGAGCAAGGGCGAGGAGCTGTTC
757	GACAAGCAGAAGAACGGCATCAAG
758	TTGATGCCGTTCTTCTGCTTGTCCGATACAGTCAACTGTCTTTG
769	GTTCGATAGAAGACAGTAGCTTCATGGTGAATTCCTCCTGCTAGG
809	ATGGAATTCGAGCTCGGTACCCGCGTTCTAGCAGGAAACAGCTATGAAGCTACTGTCTTCTATC
1045	GTGAGTTTTCGTTCCACTAGGGATAACAGGGTAATGGGATCCGATATCTAGAGGTC
1046	GTACGTACTATCAACAGGTTCCAAGCTAGCGTTGTAAAACGACGGCCAG
1259	TTTTCGTTCCACTAGGGATAACAGGGTAATCCAAGCTAGC
1260	GTACTATCAACAGGTTCCAAGCTAGCTTGGATTACCCTGT

Table S4: Primer and templates used for construction of fusion proteins.

	**λcI**	**Gal4**	**Full length mVenus**	**N-terminal fragment mVenus**	**C-terminal fragment mVenus**	**Acceptor vector**
**pMA275 (λcI-mVenus)**	646 + 656 (pPS19)	-	-	268 + 47 (pBad24-LacI-venus)	46 + 656 (pBad24-LacI-venus)	pWM2060 *Sma*I
**pMA276 (Gal4-mVenus)**	-	809 + 712 (pGBKT7)	714 + 656 (pMA275)	-	-	pWM2060 *Sma*I
**pMA270 λcI-VN + Gal4-VC)**	646 + 267 (pPS19)	270 + 758 (pGBKT7)	-	268 + 769 (pBad24-LacI-venus)	757 + 656 (pMA275)	pWM2060 *Sma*I
